# Predictors of Contemporary under-5 Child Mortality in Low- and Middle-Income Countries: A Machine Learning Approach

**DOI:** 10.3390/ijerph18031315

**Published:** 2021-02-01

**Authors:** Andrea Bizzego, Giulio Gabrieli, Marc H. Bornstein, Kirby Deater-Deckard, Jennifer E. Lansford, Robert H. Bradley, Megan Costa, Gianluca Esposito

**Affiliations:** 1 Department of Psychology and Cognitive Science, University of Trento, 38068 Rovereto, Italy; andrea.bizzego@unitn.it; 2 School of Social Sciences, Nanyang Technological University, Singapore 639798, Singapore; GIULIO001@e.ntu.edu.sg; 3 Eunice Kennedy Shriver National Institute of Child Health and Human Development, Bethesda, MD 20892, USA; marc.h.bornstein@gmail.com; 4 Institute for Fiscal Studies, London WC1E 7AE, UK; 5 UNICEF, New York, NY 10038, USA; 6 University of Massachusetts Amherst, Amherst, MA 01003, USA; kdeaterdeck@umass.edu; 7 Sanford School of Public Policy, Duke University, Durham, NC 27708, USA; lansford@duke.edu; 8 T. Denny Sanford School of Social and Family Dynamics, Arizona State University, Tempe, AZ 85287, USA; robert.bradley@asu.edu (R.H.B.); mecosta@asu.edu (M.C.); 9 Lee Kong Chian School of Medicine, Nanyang Technological University, Singapore 636921, Singapore

**Keywords:** child development, child mortality, machine learning, education, big data

## Abstract

Child Mortality (CM) is a worldwide concern, annually affecting as many as 6.81% children in low- and middle-income countries (LMIC). We used data of the Multiple Indicators Cluster Survey (MICS) (N = 275,160) from 27 LMIC and a machine-learning approach to rank 37 distal causes of CM and identify the top 10 causes in terms of predictive potency. Based on the top 10 causes, we identified households with improved conditions. We retrospectively validated the results by investigating the association between variations of CM and variations of the percentage of households with improved conditions at country-level, between the 2005–2007 and the 2013–2017 administrations of the MICS. A unique contribution of our approach is to identify lesser-known distal causes which likely account for better-known proximal causes: notably, the identified distal causes and preventable and treatable through social, educational, and physical interventions. We demonstrate how machine learning can be used to obtain operational information from big dataset to guide interventions and policy makers.

## 1. Child Mortality Is (Still) a Worldwide Concern

Nearly 4% of the world’s children each year do not survive to their fifth birthday [1]. Moreover, a large, growing, and worrisome disparity in Child Mortality (CM) exists between Low- and Middle-Income Countries (LMIC) and high-income countries. In high-income countries, advances in healthcare, access to clean water and sanitation, and improvements in overall quality of life have reduced the under-5 mortality rate to 5 per 1000 live births [2]. These life enhancing conditions stand in contrast to conditions present in LMIC, where CM in 2018 was as high as 6.81% [2]. Even in LMIC that enjoy improved conditions, vast disparities in risk and protective factors and mortality rates remain depending on household circumstances and locality. Reducing CM is an explicit goal of the United Nations Development Program [3], which advocates for improvements in deleterious conditions that increase the likelihood of CM and for increased research to advance knowledge about which specific conditions are implicated in CM in different contexts. The global community recognizes the crucial need to end preventable child deaths, making it an essential part of the Global Strategy for Women’s, Children’s, and Adolescents’ Health (2016–2030) [4] and UN Sustainable Development Goal 3 [5]. In consequence, having evidence-based indications of specific factors (or combinations of factors) that carry the most weight in determining under-5 CM would contribute directly to designing tailored and more effective interventions.

Under-5 CM has both proximal and distal causes. Proximal causes are usually medical or biological conditions that result in human demise (i.e., preterm birth complications, pneumonia, congenital anomalies, sepsis, diarrhea and malaria [6]). By the time a proximal cause of death is identified, it may be too late to save a child’s life. Much attention has rightly focused on proximal causes of CM over the years. However, proximal causes arise from known and unknown distal causes. On this account, the global heath community should be equally concerned with identifying distal causes that likely give rise to proximal causes of CM. Often, distal causes are treatable or even preventable obviating proximal causes. For example, diarrhea is a common proximal cause of CM, but poor sanitation or contaminated water are major distal causes of the proximal diarrhea cause of mortality in young children [7].

Multiple potential distal causes have been identified, but research so far has focused on only a small number of distal causes at time. These restricted approaches help in understanding how and why a distal cause is associated with CM, but have inhibited identifying the relative importance of different causes. Organizations and institutions need to know which causes are more relevant and should be targeted first.

The primary aim of this study was to identify and order by predictive power distal causes that contribute to under-5 CM. To reach this aim, we applied Machine Learning (ML) to a large multivariate UNICEF dataset from a large number of LMIC.

## 2. Materials and Methods

In this study we used data from the Multiple Indicator Cluster Survey (MICS), an international household survey developed and supported by UNICEF. We selected and processed the dataset to obtain information about mothers and the environment in which they lived (see *Participants*). In particular, we selected MICS items that inform about distal causes potentially associated with CM.

We then applied a ML approach to automatically identify mothers who had at least one child who died before the age of 5 years, based on selected MICS items (see *Predictive Performance of MICS Indicators for Under-5 Child Mortality*).

From the trained model, we obtained the importance of the MICS items in terms of predictive potency, and analysed the top 10 items in terms of relevance to proximal causes of CM. Finally (see *Predictive Confirmation of MICS Indicators in Reducing Under-5 Child Mortality*), we retrospectively validate the results, by analysing how country-level variations of CM are associated with the variation of the percentage of households with improved conditions, across two administrations of the MICS.

### 2.1. Participants

The sample consisted in 275,160 mothers between the ages of 15 and 49 years (M = 32.85, SD = 8.44) from 247,247 households that were randomly selected from 27 LMIC (for the selection of the households see Section 1: MICS in Supplementary Material). Mothers were classified as (a) mothers with no deceased child before the age of 5 years (noU5D) and (b) mothers with at least one child who died before the age of 5 years (U5D). The noU5D class could include mothers with a child who died after the age of 5 years. Data were extracted from the 2009–2013 and 2013–2017 (round 4 and 5) administrations of the MICS. All MICS indicators were screened, and 37 (see Table 1) were selected as pertinent for this study (for the selection procedure see Section 2: Data processing in Supplementary Material). Ethics approvals were handled in each site in which data were collected.

### 2.2. Predictive Performance of MICS Indicators for under-5 Child Mortality

**Figure 1:**
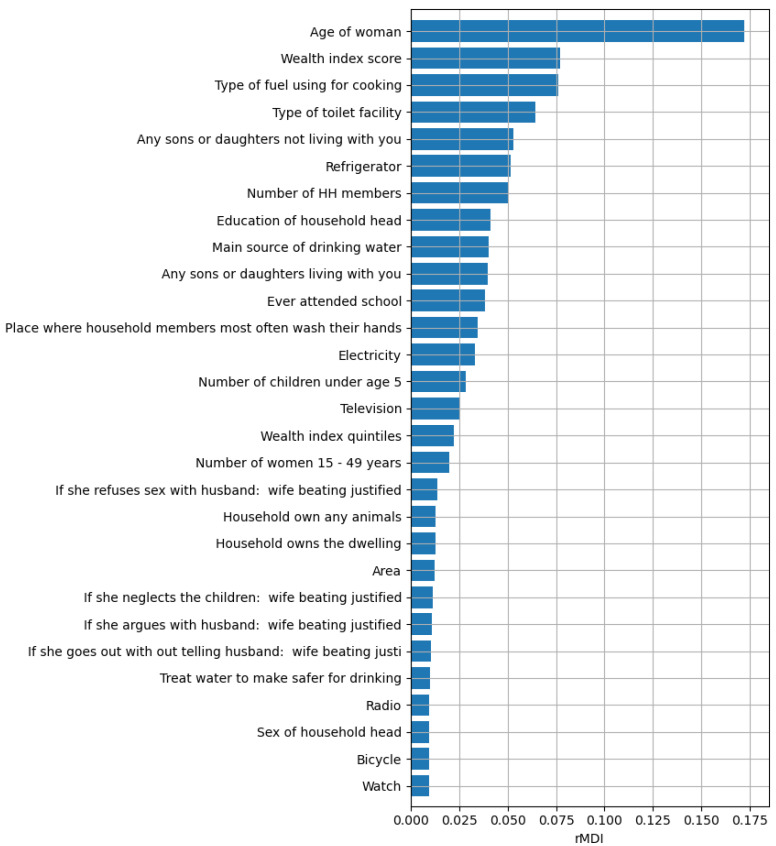
The 29 most important MICS indicators ranked according to the relative Mean Decrease Impurity (rMDI) metric.

In this study we leverage on a ML model to extract information from the MICS dataset, similar to other data mining approaches [8,9]. A Random Forest model [10], which is based on an ensemble of decision trees, was trained on the 37 MICS indicators to predict mother class (U5D v. noU5D) and identify indicators associated with CM. Having assessed the reliability of the model, we considered the importance of MICS indicators resulting from the training process (for the model optimization and stability see Section 3: Analytical Plan of Supplementary Material). The ranked list of 29 residual indicators is shown in Figure 1.

**Figure 2:**
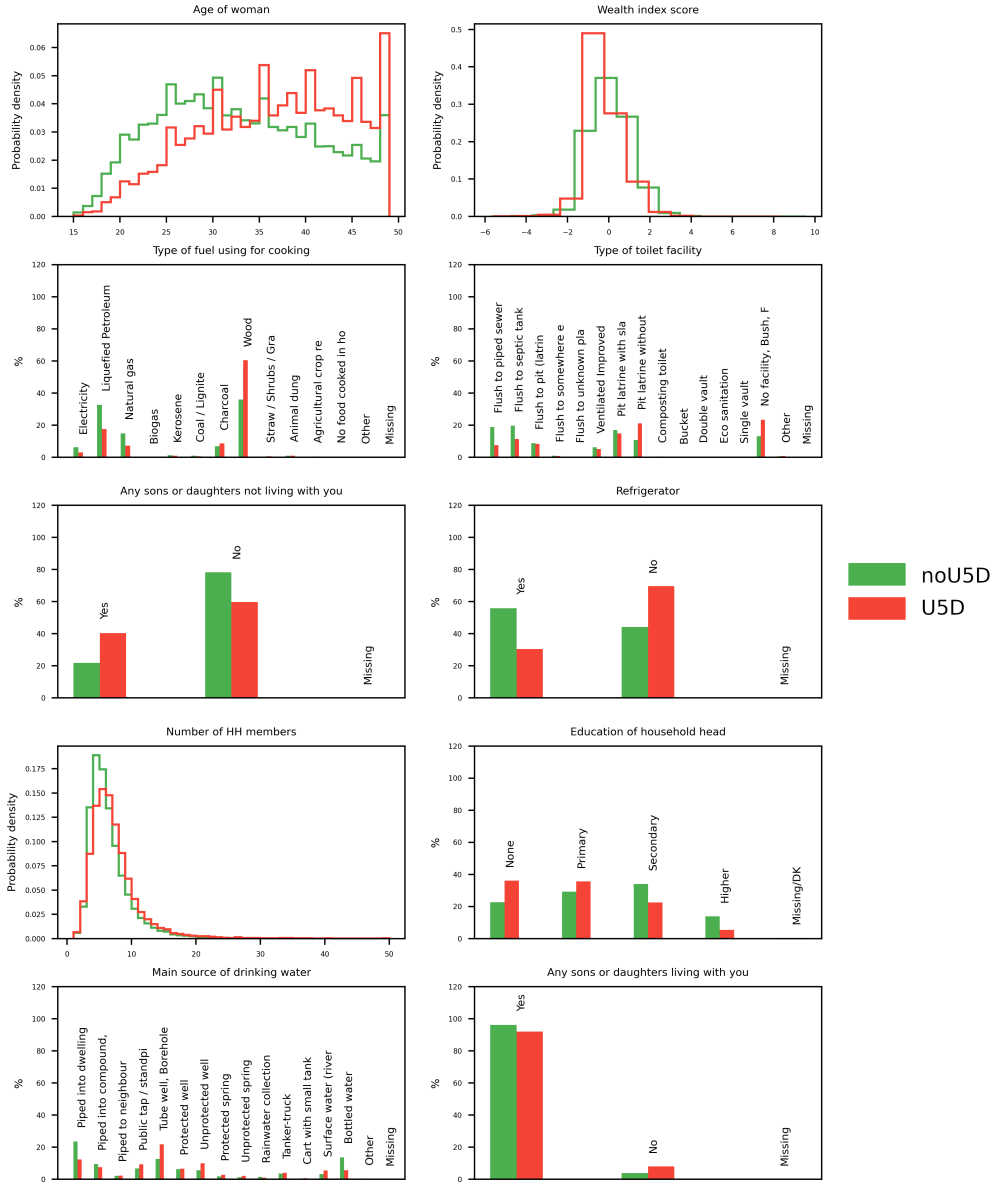
Descriptive distribution of the values of the top ten MICS predictors of Child Mortality (CM) in the two mother groups: noU5D (black) and U5D (gray). Nominal indicators are reported as percentage of the total number of mothers in the group. Numerical indicators are reported as histograms of the probability density.

This ML analysis of 37 potential distal causes of under-5 CM in more than a quarter of a million households in 27 LMIC identified and prioritized the top 10 likely distal causes, whose distributions are shown in Figure 2 (for the analysis of ML results see Section 4: Machine Learning Results of Supplementary Material).

The top 10 indicators naturally fall into 3 groups: 2 (#1 and #8) refer to characteristics of the mother or head of household, with older maternal age (rMDI = 0.172) and education of the head of the household (rMDI = 0.041) associated with greater likelihood of having had a child who died before the age of 5. Five of the top 10 predictors of CM refer to characteristics of the home environment, including #2 family wealth index score (rMDI = 0.077), #3 fuel used for cooking (rMDI = 0.076), #4 toilet facilities (rMDI = 0.064), #6 availability of refrigeration (rMDI = 0.052), and # 9 drinking water (rMDI = 0.04). Three personal characteristics of the household complete the top ten: #5 other (living) children not living in the household (rMDI = 0.053), #7 the number of household members (rMDI = 0.051), and #10 the number of children living in the household (rMDI = 0.04).

### 2.3. Predictive Confirmation of MICS Indicators in Reducing under-5 Child Mortality

The data set used to identify the top 10 distal causes of CM came from the 2009–2013 and 2013–2017 administrations of the MICS. To confirm the efficacy of these 10 distal causes in reducing CM, we used data from the 2005–2007 administration (round 3) of the MICS for 7 countries which took part in both the 2005–2007 and 2013–2017 MICS administrations and computed both the improvement in each distal cause and the change in CM between the two administrations (see Section 3: Analytical Plan in Supplementary Material).

**Figure 3:**
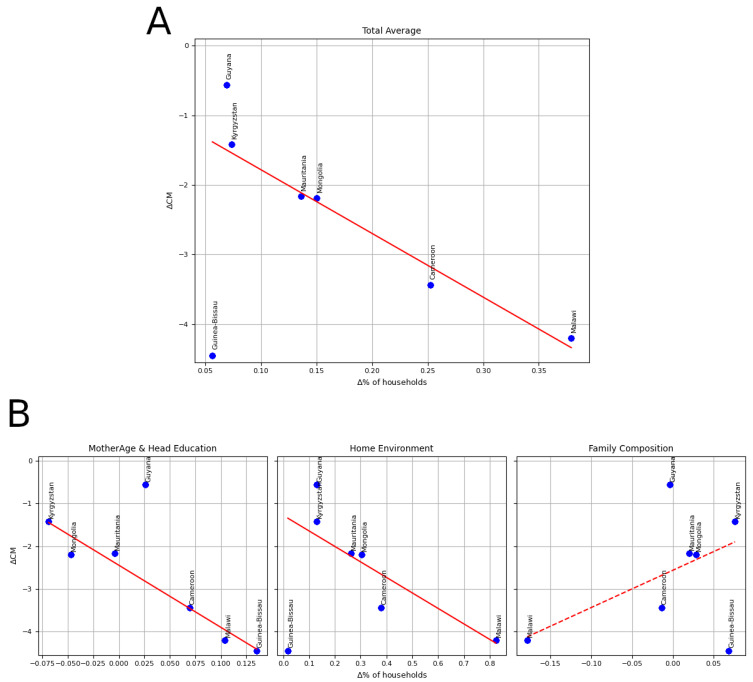
Association between the improvement of distal causes and CM. ΔCM: variation of the incidence of Child Mortality between round 3 and 5; Δ%: variation of the percentage of households with improved characteristics. (**A**). Total Improvement; (**B**). Three groups of distal causes: Mother Age and Head Education (left), House Environment (middle), and Family Composition (right). Solid lines indicate a significant association, according to the linear regression models (Table 2); dashed lines indicate non significant association.

Next, we estimated the relevance of each group of distal causes in reducing CM [11] by fitting a linear regression to predict the variation in CM (ΔCM) based on the variation of the percentage of households with improved characteristics of the identified distal causes (Δ%). Confirming the ML analysis, significant linear associations emerged for the first two groups of predictors and for the total improvement (Figure 3 and Table 2).

## 3. Distal Causes of Child Mortality

Notably, all 10 likely distal causes of CM are preventable and treatable using social, educational, or physical interventions. The overall improvement in the top 10 distal causes is significantly associated with the reduction of CM (p<0.00001). Here we discuss the top 10 distal causes, considering the three groups: characteristics of the mother and household head, the home environment, and household composition.

### 3.1. Characteristics of the Mother and Household Head

The improvement in the characteristics of the mother and household dead was significantly associated with the reduction in CM (p<0.00001).

#### 3.1.1. Older Maternal Age

It has long been known that children born to very young (teenage) mothers are at increased risk of poor health outcomes and even death [12], and that pregnancy timing associated with the healthiest outcomes for children occurs in mothers’ 20 s [13]. We found that older maternal age was the strongest predictor of having had a child younger than 5 die. One interpretation is that older mothers have a greater probability of having more children and, as a consequence, greater probability of having a child who died before the age of 5. Alternately, there is greater risk for both maternal and CM at very old (as there is at very young) ages of mothers [14]. Some MICS data support the first interpretation; two Pearson correlations (N = 644,009) showed associations between mothers’ age and their number of children, r=0.57,p<0.001, and number of children and number of deceased children, r=0.55,p<0.001. However, on the second interpretation it is also the case that mothers over 35 years are more likely to have preterm births, and intrauterine growth retardation and being born low birth weight are also more likely for children born to mothers over the age of 35 [15]. Rates of chromosomal disorders and prematurity in offspring to older mothers are much greater than those to younger mothers [15] as are congenital malformations [16] and multiple births [17]. For women over 50, these problems multiply [18]. Whichever or both, our results (based on data going up to age 5 years) draw attention to the lesser recognized fact that, after infancy and in the aggregate, older maternal age is a strong predictor of having a child under-5 die.

#### 3.1.2. Education of the Household Head

Children of mothers who lack any education are 2.6 times more likely to die before reaching age 5 compared to children of mothers with a secondary or higher education [19]. Mothers’ education correlates significantly with nutritional and developmental gains of their children [20]. Parent educational level predicts knowledge of child care and child development [21].

### 3.2. The Home Environment

The improvement in the Home Environment was significantly associated with the reduction in the CM (*p* = 0.03919).

Scientists, policy makers, and humanitarians have long pointed to housing quality and its effects on human well-being [22], including direct links to CM [23]. Five of the top 10 predictors of CM involve characteristics of the home environment, including the family wealth index score, fuel used for cooking, toilet facilities, refrigeration, and quality of drinking water.

#### 3.2.1. Family Wealth

Along with education and occupation, family wealth is a central component of socio-economic status [24]. Socio-economic status is associated with differences in parenting behaviours that are connected with nutrition and health promotion, such as prenatal care and breastfeeding, beginning very early in a child’s life [25]. Children with low socio-economic status are more likely to suffer injuries and to die, even in high-income countries [26], as their homes often have safety hazards (e.g., water heaters set too high in temperature) and lack safety protections (e.g., smoke alarms; [27]). Likewise, poor families are likely to live in places that increase children’s exposure to pollutants and neurotoxins, with negative implications for health and survival [28]. Under-5 mortality rates are, on average, twice as high for the poorest households in LMIC compared to the richest [29].

#### 3.2.2. Cooking Fuel

Not having electricity imposes problems on health as families are forced to resort to solid biofuels to cook or to provide heat. Such devices often produce smoke that is not properly ventilated, and resultant high levels of indoor pollutants contribute to premature child death. Having an open stove with no chimney in the household increases indoor pollutants that pose health risks, just as open fires in a house increase susceptibility to respiratory illness [30]. In LMIC, acute respiratory illness is a leading proximal cause of death among young children, but acute respiratory illness is associated with exposure to indoor air pollution [27]. Exposure to wood smoke has other adverse health consequences for children as well. Inadequate ventilation increases the likelihood of childhood asthma, respiratory infections, and inflammations. The World Health Organization estimated that 2.3 billion people in LMIC use biomass fuels or coal for cooking, particularly those living in rural areas with limited access to electricity [31].

#### 3.2.3. Toilet Facilities

Not having proper facilities to deal with waste contributes to childhood illness and mortality [32]. To reduce CM, it is critical to eliminate children’s exposure to excreta. In rural areas around the world, less than half the population has access to improved sanitation facilities [33]. In some informal urban settlements in poor Asian, African, and South American cities, mortality rates among young children are estimated at 150–200 per 1000 [34] where the largest number of cases involve diarrhea, which is caused by bacterial, viral, and parasitic pathogens connected to poor hygiene and sanitation [35].

#### 3.2.4. Refrigeration

Food-borne illnesses are common when homes lack adequate facilities for food preparation and storage, which often means food is left out for later consumption, thus increasing the likelihood of contamination [36]. Many pathogens multiply more quickly at room temperature than when refrigerated or frozen, which creates a risk for food contamination and gastro-intestinal illness [37]. Cooking foods often kills bacteria in the short term, but microbes quickly multiply when cooked foods are not properly stored. Contamination of foods used to wean the child from the breast is a leading cause of diarrheal disease and malnutrition in children under 5 [38]. Thus, having facilities for properly storing and refrigerating food is critical to reducing disease exposure.

#### 3.2.5. Drinking Water

Children without access to clean water are at much higher risk of diarrheal diseases and for waterborne diseases, such as cholera and enteric fevers. Lack of access to clean water is also a major contributor to poor nutritional status [39], and diarrhea and intestinal parasites contribute to malnutrition [40]. Data from 74 countries revealed that under-5s were most likely to live in houses that had no access to clean water [41]. Even when communities or countries make provisions for improving access to drinking water, the source of water and the manner in which water is transported undermine children’s health. Children’s vulnerability to pathogens from contaminated water relates to their exposure: Children actively explore their environments and are unaware of sanitation conditions that can lead to morbidities through disruptions in the immune system. When water does not come into the house, storage becomes a contamination issue. Young children may dip their hands into water or drop water scoops on the floor, which then becomes a source of disease [42].

### 3.3. Household Composition

Three characteristics of household composition complete the top 10 determinants of CM we identified. They are other (living) children not living in the household, the number of household members, and the number of children living in the household. Perhaps the second two relate commonly to pernicious generic effects of crowding in the household and, in combination with the first, to the division of finite parenting resources.

The association between improvement of Household Composition and reduction in CM was not significant (*p* = 0.10907): this might be due to the fact that these distal causes require more time to show their effects on the reduction in CM or because distal factors can work and combine in a variety of ways to promote a particular outcome, sometimes including trade-offs with other distal factors. The untoward consequences of crowding have long been of concern to scholars interested in the health and adaptive functioning of children [43]. Crowded conditions are systematically associated with respiratory illnesses, meningitis, and gastrointestinal problems in children [44]. Crowding encourages the spread of infection and increases the likelihood of injury and death as a consequence of lifestyle choices, like co-sleeping which may be a factor in sudden infant death syndrome [45]. Living in crowded quarters increases the likelihood of poor overall health among children, partly as a consequence of having more direct exposure to viral and bacterial pathogens [44].

Overcrowding often coincides with poor overall housing quality (e.g., poor ventilation, lack of household facilities, poor external construction) [46]. Living in confined space increases stress and poor treatment by others in the household: For example, parents living in crowded conditions are less responsive to and involved with their children and engage in less effective monitoring and lower levels of individual supervision [47]. Age spacing in the household composition could also have an impact in terms of (a) what types of pathogens a young child is likely to be exposed to, and (b) what type of care a young child is likely to receive [48]. In effect, the overall impact of crowding could reduce the likelihood that parents engage in behaviors that reduce the probabilities of children being injured or becoming ill.

## 4. Implications for Intervention

The pressing question is often asked: “What effective and sustainable interventions promote and support behavior changes required to accelerate reductions in under-5 mortality?” The high-ranking distal predictors of CM identified in this study point to optimal targets to pursue to reduce CM. Thus, the findings may help governments and NGOs in their humanitarian campaigns. Our findings suggest that many under-5 child deaths are likely preventable by attending to distal their causes. Young children who die each year could be saved by discouraging “geriatric primiparity,” improving education of household heads, augmenting family wealth, improving housing (focusing on cooking fuels, toilet facilities, refrigeration, and drinking water), and reducing family size and crowding in the home. For example, an important caregiver behavior of that contributes to child survival is knowledge of the correct infant sleeping position. In Argentina and Brazil, educational interventions that encouraged caregivers to put their infants to sleep on their backs resulted in significant improvements in sleeping position and reduced sudden infant death syndrome [49]. Of course, such interventions constitute only one part of a comprehensive strategy for enhancing child survival. They need to be complemented with interventions targeting communities and health care systems. For example, a prominent barrier to remediating CM is the belief that only high technology, clinic-based interventions can reduce mortality [50]. Health care workers may believe that only intravenously delivered solutions combat diarrhea and that this treatment is expensive, requires skilled care, and is not readily available to poor mothers in LMIC. Although effective oral rehydration salts may be available at minimal cost, the causal factors we have identified upstream from downstream treatments may obviate diarrhea in the first place. In short, our findings have two noteworthy policy implications. First, initiatives to increase public awareness about the roles of age and education as well as specifics of the household environment and composition offer promise to reduce CM. Second, the current study might promote interest in a “big data” approach to other topics in the social and behavioral sciences as well as public health.

## 5. Conclusions

In this study we used a machine learning framework derived from bio-informatics to delineate and rank distal causes of under-5 CM in LMIC. To our knowledge, this is the first time that a ML framework has been applied to the MICS dataset or to identifying and prioritizing causes of under-5 CM.

By 2017, 118 of 195 countries had an under-5 mortality rate below the UN Sustainable Development Goal target of fewer than 25 deaths per 1000 live births. Progress will need to accelerate in the remaining 77 countries (many in sub-Saharan Africa and Southern Asia) to achieve the target by 2030 [51]. In addition, in countries that have already achieved the target, efforts should be intensified to reduce within-country inequity in mortality. When the knowledge and technology for life-saving interventions are available, it is unacceptable that 15,000 children die every day from preventable causes. Our study identifies and priorities up-stream distal causes—and presumptive targets of intervention—likely to reduce down-stream proximal causes of under-5 CM.

Mortality rates among children are key indicators of child well-being and more broadly of future sustainable social and economic development. CM is part of the larger canon of health disparities that pervade communities and nations with poor resources and that are at the forefront of efforts by international organizations, such as the United Nations and the World Health Organization, to improve human life [3]. Identifying factors that influence CM is requisite to evidence-based policymaking to improve the survival chances of the world’s children.

Although knowledge about proximal causes of CM has advanced considerably over the last half-century, less research has been done to understand the distal causes of those proximal causes, especially in LMIC. Our understanding of child survival, derived from research conducted in high-income countries, may not readily apply to LMIC. The poverty and limited resources that characterize environments in LMIC present difficult circumstances for research, policies, and programs that promote child well-being. Many families living in LMIC experience physical and social conditions that increase the likelihood children will not live to see their 5th birthday. The present study identifies and orders distal causes of under-5 CM in a large number of LMIC. The results point to concrete protective factors that can be addressed so that more children will survive. Innovative analytic methods, such as those used in this study, will help to move that process forward.

## Figures and Tables

**Table 1:** Multiple Indicators Cluster Survey (MICS) items used in this study. HH: Household questionnaire, WM: Women’s questionnaire.

Module	MICS Item	Description
HH	HC6	Type of fuel using for cooking
HH	HC8A	Electricity in the Household
HH	HC8B	Household owns: Radio
HH	HC8C	Household owns: Television
HH	HC8D	Household owns: Landline Telephone
HH	HC8E	Household owns: Refrigerator
HH	HC9A	Household owns: Watch
HH	HC9B	Household owns: Mobile telephone
HH	HC9C	Household owns: Bicycle
HH	HC9D	Household owns: Motorcycle or scooter
HH	HC9E	Household owns: Cattle/Donkey/Horse Cart
HH	HC9F	Household owns: Car or truck
HH	HC9G	Household owns: Boat with motor
HH	HC10	Household owns the dwelling
HH	HC13	Household owns any animals
HH	HC15	Any household member own bank account
HH	HH6	Area
HH	HH11	Number of HH members
HH	HH12	Number of women 15–49 years
HH	HH14	Number of children under age 5
HH	hHighEL	Education of household head
HH	HHSEX	Sex of household head
HH	HW1	Place where household members most often wash their hands
HH	windex5	Wealth index quintile
HH	WS1	Main source of drinking water
HH	WS6	Treat water to make safer for drinking
HH	WS8	Type of toilet facility
HH	wscore	Combined wealth score
WM	CM4	Any sons or daughters living with you
WM	CM6	Any sons or daughters not living with you
WM	DV1A	If she goes out without telling husband: wife beating justified
WM	DV1B	If she neglects the children: wife beating justified
WM	DV1C	If she argues with husband: wife beating justified
WM	DV1D	If she refuses sex with husband: wife beating justified
WM	DV1E	If she burns the food: wife beating justified
WM	WB2	Age of woman
WM	WB3	Ever attended school

**Table 2:** Results coefficients of the linear regression to predict the variation in CM (ΔCM) based on the variation of the percentage of households with improved characteristics of the identified distal causes (Δ%).

Group	Linear Coefficient	*p*-Value
Total Improvement	−9.17	<0.00001
Mother Age & Head Education	−14.54	<0.00001
Home Environment	−3.63	0.03919
Family Composition	8.73	0.10907

## Data Availability

Data can be obtained from UNICEF at mics.unicef.org.

## Supplementary Materials

The following are available online at https://www.mdpi.com/1660-4601/18/3/1315/s1.
