# Supplementary material for: Predictors of Contemporary under-5 Child Mortality in Low- and Middle-Income Countries: A Machine Learning Approach
Source: Int J Environ Res Public Health. 2021 Feb 1;18(3):1315. doi: 10.3390/ijerph18031315 (PMC7908191; doi:10.3390/ijerph18031315)
Supplement: Supplementary file 1 [file ijerph-18-01315-s001.pdf]

International Journal of
Environmental Research and Public Health

MDPI

Article

Predictors of Contemporary under-5 Child Mortality in Low- and Middle-Income Countries: A Machine Learning Approach - *Supplementary Material*

Andrea Bizzego [1], Giulio Gabrieli [2], Marc H. Bornstein [3,4,5], Kirby Deater-Deckard [6], Jennifer E. Lansford [7], Robert H. Bradley [8], Megan Costa [8] and Gianluca Esposito [1,2,9,*]

1 Department of Psychology and Cognitive Science, University of Trento, Rovereto, 38068, Italy; andrea.bizzego@unitn.it
2 School of Social Sciences, Nanyang Technological University, Singapore, 639798, Singapore; GIULIO001@e.ntu.edu.sg
3 *Eunice Kennedy Shriver* National Institute of Child Health and Human Development, Bethesda, MD, 20892, USA; marc.h.bornstein@gmail.com
4 Institute for Fiscal Studies, London, WC1E 7AE, UK
5 UNICEF, New York, NY, 10038, USA
6 University of Massachusetts Amherst, Amherst, MA, 01003, USA; kdeaterdeck@umass.edu
7 Sanford School of Public Policy, Duke University, Durham, NC, 27708, USA; lansford@duke.edu
8 T. Denny Sanford School of Social and Family Dynamics, Arizona State University, Tempe, AZ, 85287, USA; robert.bradley@asu.edu (R.H.B.); mecosta@asu.edu (M.C.)
9 Lee Kong Chian School of Medicine, Nanyang Technological University, Singapore, 636921, Singapore
* Correspondence: gianluca.esposito@ntu.edu.sg or gianluca.esposito@unitn.it

check for updates

Citation: Bizzego, A.; Gabrieli, G.; Bornstein, M.H.; Deater-Deckard, K.; Lansford, J.E.; Bradley, R.H.; Costa, M.; Esposito, G. Predictors of Contemporary under-5 Child Mortality in Low- and Middle-Income Countries: A Machine Learning Approach - *Supplementary Material*. *Int. J. Environ. Res. Public Health* **2021**, *1*, 0. https://doi.org/

Academic Editor:Keun Ho Ryu
Received:
Accepted:
Published:

Publisher's Note: MDPI stays neutral with regard to jurisdictional claims in published maps and institutional affiliations.

cc BY

Copyright: © 2021 by the authors. Licensee MDPI, Basel, Switzerland. This article is an open access article distributed under the terms and conditions of the Creative Commons Attribution (CC BY) license (https://creativecommons.org/licenses/by/4.0/).

Abstract: Child Mortality (CM) is a worldwide concern, annually affecting as many as 6.81% children in low- and middle-income countries (LMIC). We used data of the Multiple Indicators Cluster Survey (MICS) (N = 275,160) from 27 LMIC and a machine-learning approach to rank 37 distal causes of CM and identify the top 10 causes in terms of predictive potency. Based on the top 10 causes, we identified households with improved conditions. We retrospectively validated the results by investigating the association between variations of CM and variations of the percentage of households with improved conditions at country-level, between the 2005–2007 and the 2013–2017 administrations of the MICS. A unique contribution of our approach is to identify lesser-known distal causes which likely account for better-known proximal causes: notably, the identified distal causes and preventable and treatable through social, educational, and physical interventions. We demonstrate how machine learning can be used to obtain operational information from big dataset to guide interventions and policy makers.

Keywords: child development; child mortality; machine learning; education; big data

1. MICS

1.1. MICS sampling

The selection of the target households follows a three-stages process. The first-stage, or primary sampling units, is defined, if possible, as census enumeration areas. The second stage is the selection of segments. The third stage is the selection of the particular households within each segment that are interviewed in the survey. Two countries are present in both 2009-2013 and 2013-2017 MICS administrations (MICS rounds 4 and 5): Sudan and Swaziland. As the households are randomly sampled in both rounds, we deemed that the two rounds sample different households and kept both occurrences in the dataset.

1.2. MICS structure

The MICS questionnaire is divided into modules. In this study we used data from 3 modules:

- Household module (HH): household characteristics and equipment;
- Women module (WM): woman's instruction, health, wealth, domestic violence, use of substances;
- Birth/death module (BH): birth and death of the children of the household.

Of the 106 countries available in rounds 4 and 5 of MICS, 55 did not provide the BH module. Consequently, these 55 countries were excluded from the study. To join the data of the three modules we created specific data keys:

- HHID: Household ID, unique identifier of the household. HHID is derived as a composition of the following indicators: [MICSround]_[Country]_[HH1]_[HH2], where HH1 is the identifier of the cluster and HH2 is the identifier of the household within the cluster. The HHID key is computed for each module;
- HLID: House line ID, unique identifier of a subject. HLID is derived as a composition of [HHID]_[subID], where subID is the indicator that identifies the respondent or the subject of the question: LN in WM, BH8 in BH). The HLID key is computed on all modules, with exception of HH;
- MOID: Mother ID, unique identifier of the mother of the child in the BH module. MOID is therefore computed only on BH and derived as a composition of [HHID]_[LN].

2. Data Processing

2.1. Selection of indicators and mothers

Not all the MICS indicators are available for all samples. Therefore, we had to shortlist the indicators to maximize the number of available samples and informativeness of the dataset. We manually selected 120 indicators from two MICS modules: HH and WM. The list of the 120 indicators is presented in Supplementary Table 1.

In particular, from HH we selected indicators associated with presence of home equipment (e.g. presence of a refrigerator in the household) or household characteristics (e.g. number of members living in the household). From WM we selected female respondents' demographic characteristics (e.g. if she ever attended school), health and behaviour (e.g. use of substances), and social role (e.g. questions about domestic violence). We selected only the datapoints associated with mothers. To this aim, we first obtained the HLIDs of all the mothers, which are the unique values of the MOID key of the BH module. The HLIDs of the mothers are used to select the data in the WM and HH modules. Then, we joined the WM and HH data using the shared key HHID.

We categorized mothers into two classes: a) noU5D: mothers with no child deceased before the age of 5 years and b) U5D: mothers with at least one child who died before the age of 5 years. More specifically, we computed the age at death of each deceased child in BH; if the age at death of child was below 5 years the corresponding mother was categorized as U5D.

2.2. Data Parsing and Imputation

From the original 120 indicators, 20 were multiple choice answers to five different questions. These 20 indicators were therefore transformed into 5 categorical indicators indicators representing the choice for each question. This operation reduced the number of indicators to 105. Then, there were indicators that were meaningful only in case another question had a specific answer (e.g. "Number of cigarettes smoked in the past 24 hours" is meaningful only for individuals who smoke). When not meaningful, those indicators have been set to a value representing non-defined answers, to avoid considering them as missing data.

The final part of the preliminary data processing was directed at resolving missing data issues. There were two primary reasons for missing data. One source of missing indicators derived from differences in each country's policies (e.g., a country's government might decide not to disclose part of the dataset). The second source was errors in the reporting of the answers (e.g., data losses). We note that the MICS dataset has specific annotations to describe when a

participant refused or was unable to answer a question (Missing/Does not answer), or was unable to provide a correct answer (Does not remember/Does not know). In this study, these cases are not considered as missing data and the given annotation was considered as a specific type of answer.

This strategy to deal with missing data tries to recover the data by applying a missing data imputation algorithm [1,2]. This choice of recovering missing data is motivated by the objective to increase the sample size of the dataset. However, the imputation was not performed if either of the following two criteria applied:

- The datapoint has more than 25% of indicators with missing data. In this case the datapoint is removed from the dataset;
- The indicator has more than 25% of datapoints with missing data. In this case the indicator is removed from the dataset.

In the other cases the missing data are imputed by applying the multivariate imputation algorithm [1,2], as implemented in the scikit-learn Python package (v0.21.2)[3], with default settings. As the algorithm generates the imputed data on a continuous scale, in case of ordinal/nominal indicators we round the values to the nearest integer. The imputation procedure is first applied on each country independently to maintain the country-specific statistical properties of the values of each indicator. As a result, each country has a different number of indicators: either because originally some indicators were not provided, or because some indicators were removed due to a high number of datapoints with missing data. For each indicator, we counted the number of datapoints available: of the 105 selected indicators, only the 38 with more than 200,000 datapoints were kept. The datasets of the countries were then merged; however, a country's dataset was not merged if more than 10 indicators from the target 38 indicators were missing. A second data imputation procedure was then applied on the merged dataset to recover the data of the countries with missing indicators. One indicator (WS9) was removed at this stage due to many missing data.

2.3. Results of Preprocessing

The dataset obtained after the preliminary data processing step was completed is composed of 275,160 mothers (Age M=32.85; SD=8.44) and 37 indicators. 229,405 mothers belong to the class noU5D (Age M=32.23; SD=8.4) and 45,755 to the class U5D (Age M=35.94; SD=7.98), from 27 countries. In the following paragraphs we comment on the results of the data imputation procedure. During the country-level missing data imputation (see Supplementary Table 2), 2.49% of the data have been imputed (422,931 values), with an average across countries of 2.65% (SD = 2.25%), with a maximum of 6.9% for Guyana (MICS 5th round).

A second imputation step was performed when the datasets of all the countries were merged. In this second step, 2.52% of the data were imputed (256,721 values) from 27 countries (State of Palestine was not merged due to many missing data). Each region contributed an average of 10,191.1 datapoints (SD=7,210.6) with a minimum of 2186 datapoints for Sao Tome and Principe, and a maximum of 32,254 datapoints for Iraq.

3. Analytical Plan

3.1. Random Forest Model

The predictive model is built on Random Forests (RF) [4], which are particularly suited to work with categorical variables (as MICS indicators are) and provide by design an indication of importance in terms of Mean Decrease Impurity index (MDI)[4]. MDI is a metrics adopted in decision trees classifiers to quantify the importance of a given variable to predict the target. In the scikit-learn[3] implementation of RF used in this study the MDI is given as the ratio of the total impurity of the input dataset, namely: relative MDI (rMDI). Due to unbalance in the number of samples for each mother class (noU5D: 83.4% of the samples; U5D: 16.6% of the samples), we assigned weights to the two classes (noU5D: 0.17; U5D: 0.83) to avoid a training which favours the over-represented class. To evaluate model performance we rely on the Matthews Correlation Coefficient (MCC), which is suited in case of an unbalanced dataset as it is computed from the full confusion matrix of the predictions[5,6].

3.2. Model Optimization

Three model parameters are optimized: the number of trees (T), the number of input indicators (K), and the depth of each tree (D). The optimization procedure is accomplished in two steps:

1. In the first step we find the optimal T with a grid search on a set of target values ($T \in \{10, 50, 100, 500, 1000\}$). This first step also returns the overall ranking of all the 37 indicators. The optimal T was 500;
2. In the second step we find the optimal K and D by grid search. D represents the total number of decisions in the tree, and its value should not be greater than the number of input indicators K. As K varies ($K \in \{1, 2, 3, 4, 5, 7, 8, 11, 14, 18, 23, 29, 37\}$) the values searched for D are then set proportionally to the input K: $D \in \{10\%, 25\%, 50\%, 100\%\} * K$. Values of

$D \leq 0$ were rejected. The ranking of the indicators from the first step is used to sort the selection of the input indicators, starting from indicators with higher rankings. The optimal K was found to be 29, with an optimal D of 13 (see Supplementary Figure 1).

3.3. Data Analysis Plan

The optimization and training of the model were performed following a procedure defined in accordance with the Massive Analysis and Quality Control (MAQC) Society[7]. In this procedure different partitions of the dataset are used to train, validate, and test the model to minimize the overfitting and provide more robust results. In particular, the data analytic plan followed these steps (see Supplementary Figure 2):

1. **First partition of the dataset**: The full dataset (D) is split into training (D_R) and test (D_S) datasets (50% of the samples for D_R and 50% of the samples for D_S), maintaining the stratification of the classes;
2. **10x5-fold CV**: The D_R is used for the grid-search optimization of the model parameters, within a cross-validation (CV) scheme. D_R is split again into 5 folds: in turn, one fold is used for validation (D_v) and the remaining four for training (D_r). The model is trained on D_r and evaluated on D_v. The output of a single training-evaluation step is the performance of the model on D_v (MCC_v) and the rMDI of the indicators ($rMDI_v$). This procedure is repeated 10 times, each time changing the 5 fold split. We refer to this procedure as 10x5-CV. For each combination of parameters, the results of the 10x5-CV are: the distribution of model performance, from which we compute the median MCC_v and 90% studentized bootstrap Confidence Intervals ($CI_{50\%}$), and the 50 lists of importance of the indicators, which we transform into rankings and merge into one list of indicator importance (B) by applying the Borda count algorithm [8];
3. **Model selection**: The 10x5-CV is repeatedly applied for each combination of model parameters (n_{T_i},d_i). The set of parameters with the higher median MCC_v and the respective indicator importance B are selected;
4. **Model training and test**: The final predictive model is set with the optimal parameters, trained on D_R and evaluated on D_S. The outputs are the performance on D_R ($MCC_{training}$) and on D_S (MCC_{test}).

This data analytic plan serves two main purposes:

1. Stability: the evaluation of the model performance and the computation of the importance of the indicators is performed multiple times on different partitions of the dataset. Thus, possible biases or variability in the performance which would affect the reliability of the results can be evidenced;
2. Reproducibility: the predictive performance of the model is evaluated on independent datasets: first on D_v (within the 10x5-fold CV), then on D_S to ensure that the results generalize to the population described by the datasets. Major differences in performance would give evidence that the training procedure is biased and, therefore, the results are not reproducible.

3.4. Predictive Confirmation of MICS Indicators in Reducing Under-5 Child Mortality

We estimated the efficacy of interventions targeting the identified distal causes on the reduction of CM. The outcome of each MICS indicator was recoded into a binary score: high and low quality; the recoding was based on the differences in the distributions between U5D and noU5D classes. Indicators derived from the Woman Questionnaire refer to women, while indicators derived from the Household Questionnaire refer to households.

For each MICS indicator, we computed the percentage of datapoints (women or households) with a high quality score in each country. The relative difference of the percentage between the MICS5 (or MICS4 depending on the country) and MICS3 was used to estimate the overall improvement of the indicator i (P_i): $P_i = \frac{\%_{i_{MICS5}} - \%_{i_{MICS3}}}{\%_{i_{MICS3}}}$ Then we computed the difference in the percentage of CM between the years corresponding to MICS5 and MICS3.

Seven countries, of those considered in this study, were included in both MICS rounds and were used. However, Guinea-Bissau is an outlier in several distal cases and is excluded from the following explorative analysis. Notably, Guinea-Bissau it is also the country with the greatest improvement in CM (-4.45%).

A linear model was fit to investigate the relationship between the improvement of each indicator and reduction of CM (see Figure 3). Negative coefficients were found for six indicators, three with a significant association. The exceptions are mainly associated with indicators of the "Household Composition", which are also characterized by minor improvements.

We then grouped the distal causes into the three groups: "Mother Age & Head Education" ("Age of Woman" and "Education Level of HH head"), "Home Environment" ("Wealth Index", "Fuel used for cooking", "Type of Toilet Facility", "Source of Water used for Drinking", "Refrigerator in the HH") and "Household Composition" ("Number of HH Members",

"Children Living With the mother", "Children Not Living With the mother"). The improvement of each group was estimated by computing the average improvement across the indicators composing the group.

The efficacy of the intervention for each group was estimated with a linear model between the improvement for each group and the variation in CM. A robust regression [9] was used to mitigate the effect of the outlier (Guinea-Bissau).

4. Machine Learning Results

We observed a weak over-fitting effect ($MCC_{training} > MCC_{test}$), which indicates that the model is partially learning some patterns in the data that are proper for the particular partition of the dataset used for training (D_R) and not of the general population. To ensure that it is not a symptom of any major flaws in the model, we trained a new model on a copy of D_R where the classes of the samples have been randomly shuffled and evaluated the fooled model on D_S. Since obtaining performances very different from 0 (MCC=0 is the performance of a random classifier) would immediately evidence the presence of major issues in the model (e.g. biases, batch effects), this diagnostic test is common in any rigorous ML analysis. We obtained an $MCC_{fooled} = -0.017$, which ensures that, except for the slight over-fitting, the model does not present any major flaws.

We used a robust machine learning framework derived from bio-informatics applications for bio-marker discovery. Our data analytic plan assessed the stability of the model; thus, we have increased assurance regarding the importance and independence of the predictors of CM. The framework was used to more precisely delineate and rank distal predictors of CM.

4.1. Correlation of the predictors

We investigated the pairwise correlation between the top 29 predictors (Supplementary Figure 4; then we investigated the correlation between the top 29 predictors with the target outcome (Supplementary Table 3). As we are dealing with different types of variables, different correlation metrics were used:

- Spearman correlation: between variables that were interval or ordinal;
- Correlation ratio: between nominal and interval variables;
- Point biserial: between interval and dichotomous variables;
- Phi coefficient: between dichotomous variables;
- Cramer's V in all other cases.

Bonferroni's correction was adopted to account for multiple hypotheses. The higher correlation in the top 10 predictors is between HC8E ("Refrigerator in the household") and HC6 ("Type of fuel used for cooking", correlation ratio = 0.68) and WS8 ("Type of toilet facility", correlation ratio = 0.58). HC6 and WS8 also have the higher correlation with the U5CM categories: the correlation ratio between HC6 and U5CM is 0.2, and between WS8 and U5CM is 0.2.

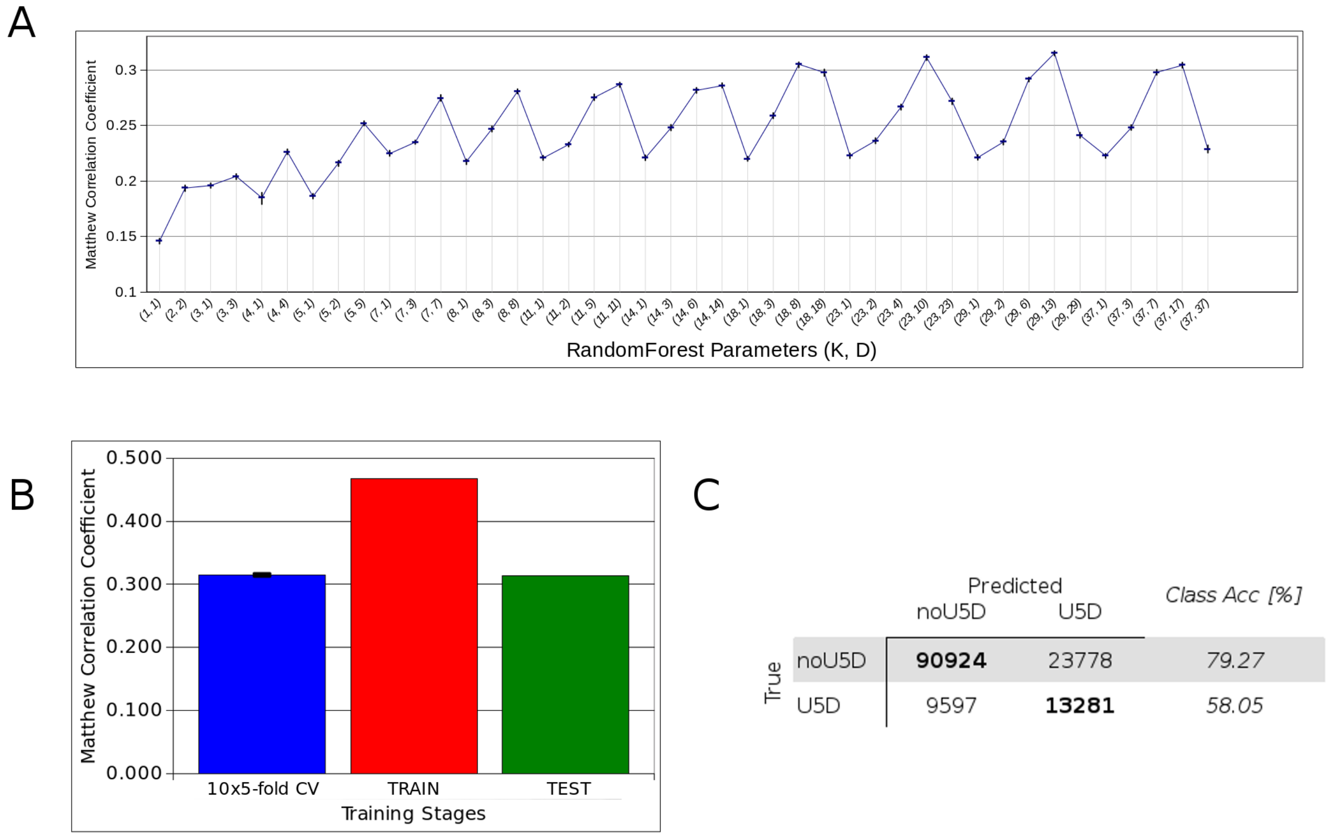

Figure 1. Predictive performances of the trained Random Forest model. A: Median MCCv and 90% studentized CI for each combination of parameters (K,D); B: Model performances for the 10x5-fold CV (best parameter set), on D_R (TRAIN) and on D_S (TEST); C: Confusion Matrix on D_S.

Table 1: Pool of the 120 indicators selected from the MICS datasets.

Acronym	Description	Acronym	Description
HH6	Area	HHSEX	Sex of household head
HH11	Number of HH members	wscore	Combined wealth score
HH12	Number of women 15 - 49 years	windex5	Wealth index quintile
HH13A	Number of men age 15-49	hHighEL	Education of household head
HH14	Number of children under age 5	WB2	Age of woman
CD3A	Took away privileges	WB3	Ever attended school
CD3B	Explained why behaviour was wrong	WB4	Highest level of school attended
CD3C	Shook child	WB7	Can read part of the sentence
CD3D	Shouted, yelled or screamed at child	MT2	Frequency of reading newspaper or magazine
CD3E	Gave child something else to do	MT3	Frequency of listening to the radio
CD3F	Spanked, hit or slapped child on bottom with bare hand	MT4	Frequency of watching TV
CD3G	Hit child on the bottom or elsewhere with belt, hair-brush, stick, etc.	MT6	Ever used a computer
CD3H	Called child dumb, lazy or another name	MT7	Computer usage in the last 12 months
CD3I	Hit or slapped child on the face, head or ears	MT8	Frequency of computer usage in the last month
CD3J	Hit or slapped child on the hand, arm or leg	MT9	Ever used internet
CD3K	Beat child up, hit over and over as hard as one could	MT10	Internet usage in the last 12 months
CD4	Child needs to be physically punished to be brought up properly	MT11	Frequency of Internet usage in the past month
HC6	Type of fuel using for cooking	cm3	Completed years since first birth
HC7	Cooking location	CM4	Any sons or daughters living with you
HC8A	Electricity	CM6	Any sons or daughters not living with you
HC8B	Radio	DB2	Wanted child later or did not want more children
HC8C	Television	CP2A	Ever used a method to avoid pregnancy
HC8D	Landline Telephone	DV1A	If she goes out with out telling husband: wife beating justified
HC8E	Refrigerator	DV1B	If she neglects the children: wife beating justified
HC9A	Watch	DV1C	If she argues with husband: wife beating justified
HC9B	Mobile telephone	DV1D	If she refuses sex with husband: wife beating justified
HC9C	Bicycle	DV1E	If she burns the food: wife beating justified
HC9D	Motorcycle or scooter	DV1F	If she neglects housework: wife beating justified
HC9E	Cattle/Donkey/Horse Cart	MA6	Marital status
HC9F	Car or truck	MA9	Age at first union
HC9G	Boat with motor	SB1	Age at first sexual intercourse
HC10	Household owns the dwelling	TA1	Ever tried cigarette smoking
HC12	Hectares of agricultural land members of household owns	TA2	Age when cigarette was smoked for the first time
HC13	Household own any animals	TA3	Currently smoking cigarettes
HC15	Any household member own bank account	TA4	Number of cigarettes smoked in the last 24 hours
WS1	Main source of drinking water	TA5	Number of days when cigarettes were smoked in past month
WS3	Location of the water source	TA6	Ever tried any smoked tobacco products other than cigarettes
WS6	Treat water to make safer for drinking	TA7	Used any smoked tobacco products during the last month
WS7A	Water treatment: Boil	TA9	Number of days when tobacco products where smoked in past month
WS7B	Water treatment: Add bleach/chlorine	TA10	Ever tried any form of smokeless tobacco products
WS7C	Water treatment: Strain it through a cloth	TA11	Used any smokeless tobacco products during the last month
WS7D	Water treatment: Use water filter	TA13	Number of days when smokeless tobacco products where used in past month
WS7E	Water treatment: Solar disinfection	TA14	Ever drunk alcohol
WS7F	Water treatment: Let it stand and settle	TA15	Age when alcohol was used for the first time
WS7X	Water treatment: Other	TA16	Number of days when at least one drink of alcohol was used in past month
WS7Z	Water treatment: DK	TA17	Number of drinks usually consumed
WS8	Type of toilet facility	LS2	Estimation of overall happiness
WS9	Toilet facility shared	LS3	Satisfaction with family life
HW1	Place where household members most often wash their hands	LS4	Satisfaction with friendships
HW2	Water available at the place for handwashing	LS5	School attendance during the current school year
HW3A	Soap or detergent present at place of handwashing	LS6	Satisfaction with school
HW3BA	Bar soap	LS7	Satisfaction with current job
HW3BB	Detergent (Powder / Liquid / Paste)	LS8	Satisfaction with health
HW3BC	Liquid soap	LS9	Satisfaction with current residence
HW3BD	Ash / Mud / Sand	LS10	Satisfaction with treatment by other people
HW5A	Soap/other material available for washing hands	LS11	Satisfaction with appearance
HW5BA	Bar soap	LS12	Satisfaction with life overall
HW5BB	Detergent (Powder / Liquid / Paste)	LS13	Satisfaction with current income
HW5BC	Liquid soap	LS14	Life satisfaction in comparison with last year
HW5BD	Ash / Mud / Sand	LS15	Life satisfaction expectation one year from now

Table 2: Summary of the by-country data imputation step. [a]: Percentage of values

Country	Round	Removed	Imputed[a]	Result #Mothers	Result #Indicators
Ghana	4	0	0.99	7696	40
Iraq	4	0	0.16	32254	72
Lao PDR	4	0	1.19	16119	45
Algeria	4	0	0.31	17636	45
Mauritania	4	0	0.41	8169	43
Moldova	4	0	0.57	4123	74
South Sudan	4	0	0.07	7322	62
Sudan	4	0	0.21	10839	75
Swaziland	4	0	0.57	3291	76
Tunisia	4	0	1.33	4479	74
Ukraine	4	0	1.90	6479	39
Cameroon	5	12	4.19	7071	74
Dominican Republic	5	426	4.52	22708	73
El Salvador	5	6	5.71	10029	73
Guinea Bissau	5	9	4.86	7523	59
Guyana	5	87	6.90	3690	53
Kosovo	5	0	0.40	3069	53
Kyrgyzstan	5	62	3.82	4986	80
Malawi	5	23	3.28	18938	74
Mali	5	0	1.09	13980	59
Mongolia	5	0	2.85	9838	41
Nepal	5	332	5.83	9630	74
Paraguay	5	129	6.64	5369	54
Sao Tome and Principe	5	4	5.01	2186	45
State of Palestine	5	681	0.03	6799	41
Sudan	5	131	3.50	11585	54
Swaziland	5	17	2.54	3276	88
Turkmenistan	5	3	5.18	4930	54
Uruguay	5	0	0.67	7101	41
Vietnam	5	7	4.90	10844	42

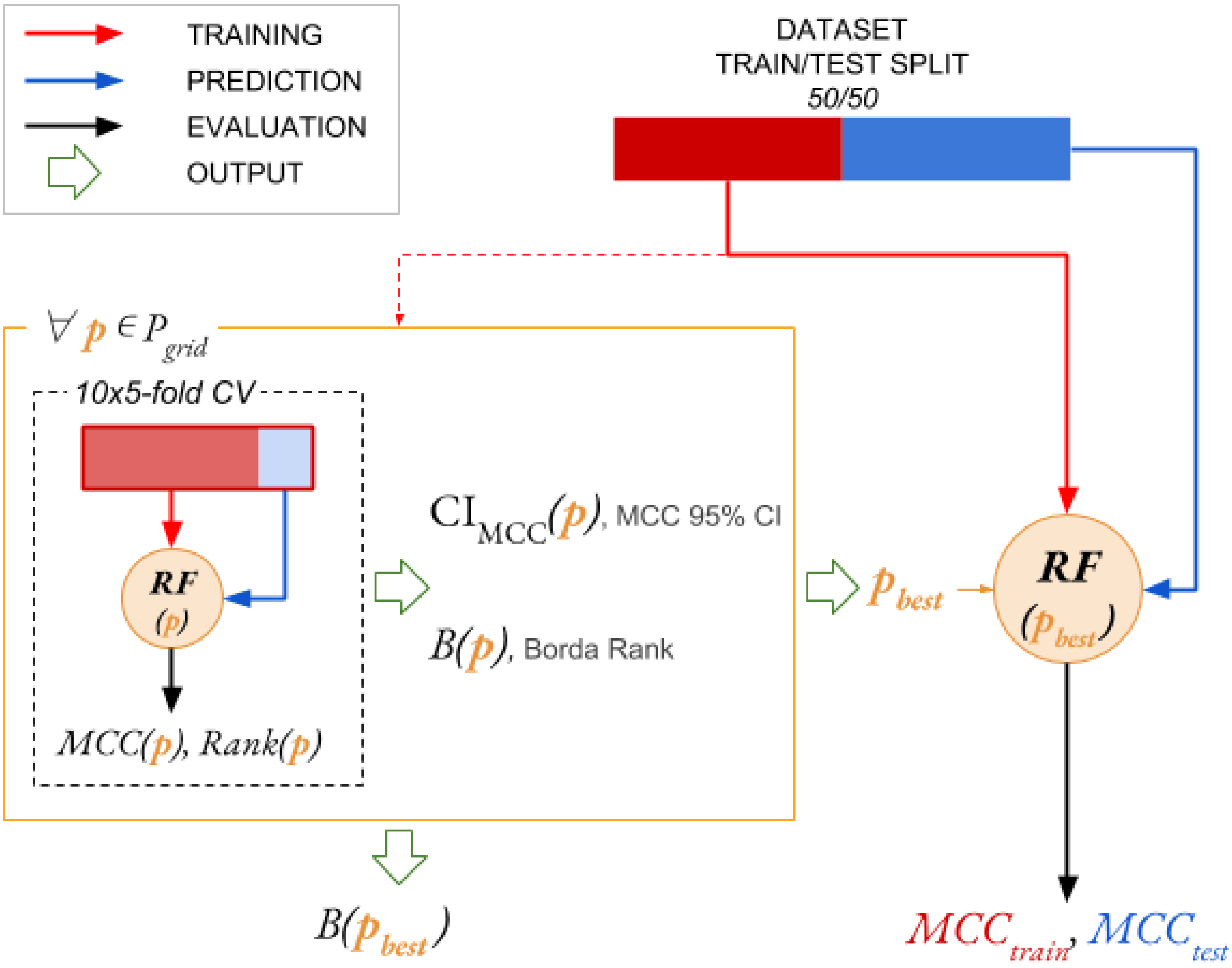

Figure 2. Schematic representation of the Data Analysis Plan.

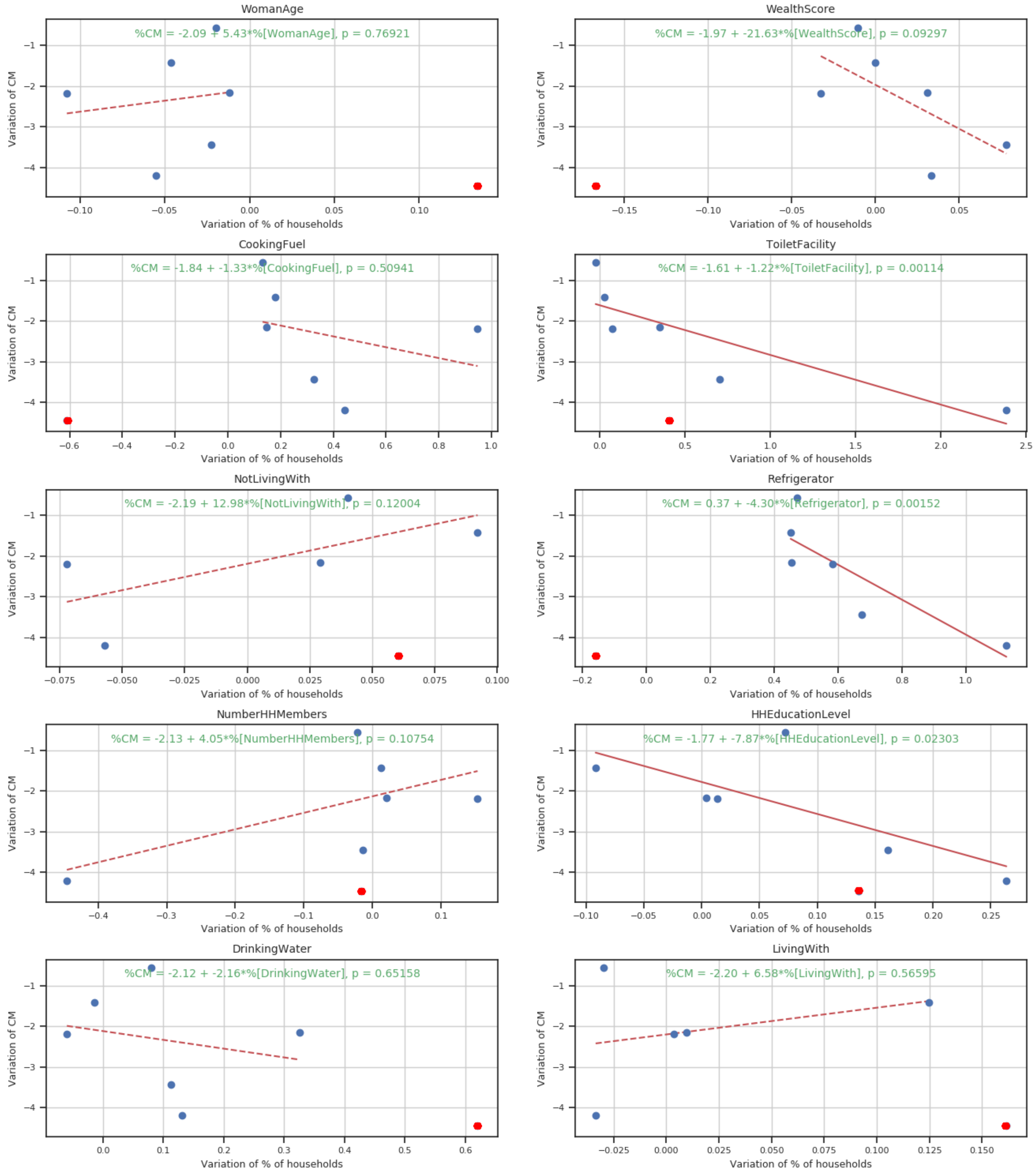

Figure 3. Association between the improvement of each MICS indicator cause and CM. The red dot indicates Guinea-Bissau, which was not used to estimate the linear models.

Rank	Acronym	Description	Variable Type	WB2	wscore	HC6	WS8	CM6	HC8E	HH11	hHighEL	WS1	CM4	WB3	HW1	HC8A	HH14	HC8C	windex5	HH12	DV1D	HC13	HC10	HH6	DV1B	DV1C	DV1A	WS6	HC8B	HHSEX	HC9C	HC9A
1	WB2	Age of woman	interval		0.08	−0.07	**0.13**	**−0.42**	**−0.15**	0.03	0.06	**0.12**	0.07	0.07	**0.12**	**−0.11**	**−0.43**	**−0.12**	0.07	**0.13**	0.02	**0.00**	−0.07	−0.06	0.01	0.01	**0.00**	−0.03	−0.01	0.03	−0.04	−0.06
2	wscore	Wealth index score	interval			**−0.45**	**0.43**	0.05	**−0.43**	−0.07	**0.40**	**0.48**	0.03	**−0.28**	**0.17**	**−0.40**	**−0.12**	**−0.42**	**0.94**	0.08	**0.13**	**0.32**	**0.22**	**−0.51**	**0.13**	**0.14**	**0.14**	−0.05	**−0.22**	0.02	**−0.14**	**−0.37**
3	HC6	Type of fuel for cooking	ordinal				**0.24**	**0.16**	**0.68**	**0.15**	**−0.38**	**0.30**	0.08	**0.32**	**0.17**	**0.63**	**0.11**	**0.61**	**−0.32**	0.01	**0.15**	**0.45**	**0.28**	**0.50**	**0.16**	**0.12**	**0.14**	**0.13**	0.09	0.07	0.08	**0.29**
4	WS8	Type of toilet facility	nominal					**0.13**	**0.58**	**0.22**	**0.23**	**0.22**	0.04	**0.28**	**0.16**	**0.64**	**0.20**	**0.57**	**0.19**	**0.12**	**0.18**	**0.40**	**0.19**	**0.47**	**0.15**	**0.13**	**0.15**	0.09	0.09	0.08	**0.12**	**0.28**
5	CM6	Children not living with you	dico						**−0.12**	0.05	0.07	**0.14**	**−0.30**	−0.08	**0.11**	**−0.11**	**0.16**	**−0.10**	0.04	−0.05	0.00	**0.10**	0.01	−0.07	−0.01	0.00	−0.02	0.01	0.03	−0.07	−0.01	−0.06
6	HC8E	Refrigerator	dico							0.09	**0.42**	**0.63**	0.03	**0.32**	**0.50**	**0.67**	**0.14**	**0.68**	**0.35**	−0.02	**−0.11**	**−0.32**	−0.09	**0.40**	−0.10	−0.08	−0.08	0.03	0.01	0.02	0.04	**0.29**
7	HH11	Number of HH members	interval								**−0.30**	**0.21**	**−0.12**	**0.24**	**0.15**	0.09	**0.44**	0.06	−0.06	**0.57**	**−0.23**	**−0.19**	**−0.16**	**0.11**	**−0.17**	**−0.17**	**−0.21**	0.03	−0.10	**−0.16**	**−0.12**	**−0.10**
8	hHighEL	Education of HH head	ordinal									**0.24**	0.03	**0.54**	**0.17**	**0.38**	**−0.18**	**0.39**	**0.37**	**−0.13**	**0.24**	**0.23**	**0.17**	**0.31**	**0.20**	**0.20**	**0.23**	**0.13**	0.03	0.04	0.06	**0.19**
9	WS1	Main source of drinking water	nominal										0.05	**0.33**	**0.15**	**0.61**	**0.18**	**0.57**	**0.22**	**0.11**	**0.23**	**0.43**	**0.28**	**0.52**	**0.20**	**0.22**	**0.22**	**0.25**	**0.14**	**0.11**	**0.20**	**0.27**
10	CM4	Children living with you	dico											−0.03	0.04	0.02	**−0.15**	0.02	0.04	−0.01	0.03	0.00	0.05	−0.01	0.03	0.03	0.04	−0.01	−0.02	0.08	0.05	0.01
11	WB3	Ever attended school	dico												**0.23**	**0.31**	**0.15**	**0.33**	**0.27**	**0.12**	**−0.27**	**−0.18**	**−0.13**	**0.23**	**−0.23**	**−0.23**	**−0.27**	0.09	0.02	−0.08	0.02	0.06
12	HW1	Place for hand washing	nominal													**0.50**	**0.15**	**0.45**	0.07	0.08	**0.14**	**0.22**	0.06	**0.26**	**0.10**	0.08	0.08	0.09	0.08	0.06	0.05	**0.24**
13	HC8A	Electricity	dico														**0.14**	**0.77**	**0.33**	−0.03	**−0.12**	**−0.30**	**−0.16**	**0.41**	−0.08	−0.10	−0.07	0.06	0.00	0.01	0.02	**0.29**
14	HH14	#children <5 yrs	interval															**0.12**	**−0.12**	**0.14**	**−0.17**	**−0.10**	−0.06	**0.11**	**−0.12**	**−0.13**	**−0.15**	0.04	−0.04	−0.10	−0.03	**0.00**
15	HC8C	Television	dico																**0.35**	−0.05	**−0.11**	**−0.28**	**−0.12**	**0.39**	−0.09	−0.10	−0.08	0.06	0.04	0.04	0.04	**0.28**
16	windex5	Wealth index quintiles	ordinal																	0.08	**0.13**	**0.28**	**0.20**	**0.47**	**0.12**	**0.14**	**0.13**	0.06	**0.23**	0.03	**0.16**	**0.33**
17	HH12	#women 15 - 49 years	interval																		**−0.12**	−0.10	−0.10	**0.00**	−0.09	−0.09	**−0.11**	**0.00**	**−0.10**	−0.01	**−0.07**	**−0.14**
18	DV1D	Beat Just: refuses sex	dico																			0.09	0.09	**−0.12**	**0.55**	**0.59**	**0.57**	−0.07	−0.02	0.09	0.00	−0.02
19	HC13	HH own any animals	dico																				**0.28**	**−0.47**	**0.10**	0.08	0.07	0.06	0.05	0.08	**0.12**	**−0.13**
20	HC10	HH owns the dwelling	dico																					**−0.31**	0.09	0.09	0.09	0.06	−0.03	0.07	0.08	−0.05
21	HH6	Area	dico																						**−0.11**	**−0.10**	−0.10	−0.01	0.04	−0.06	−0.04	**0.22**
22	DV1B	Beat Just: neglects children	dico																							**0.59**	**0.68**	−0.05	−0.05	0.08	−0.01	−0.01
23	DV1C	Beat Just: argues w/ husb.	dico																								**0.59**	−0.07	−0.05	0.07	−0.02	−0.02
24	DV1A	Beat Just: goes out w/o tell	dico																									−0.08	−0.04	**0.10**	−0.02	0.00
25	WS6	Treat water for drinking	dico																										−0.04	0.01	0.05	0.00
26	HC8B	Radio	dico																											0.03	**0.12**	**0.18**
27	HHSEX	Sex of household head	dico																												0.08	0.07
28	HC9C	Bicycle	dico																													0.08

Type of correlation	Range	Pval?	Variable Type
Spearman (red)	[-1:1]	Y	Both Interval or Ordinal
Correlation ratio (purple)	[0:1]	N	Nominal vs Interval
Point Biserial (yellow)	[0:1]	Y	Interval vs Dicothomous
Cramer's V (green)	[0:1]	N	Ordinal vs Nominal, Nominal vs Nominal, Nominal vs Dicothomous
Phi (cyan)	[-1:1]	N	Dicothomous vs Dicothomous

Figure 4. Correlations among the top 29 predictors. Colors of cells indicate the type of correlation test used. The top left box indicates the top 10 indicators considered in the validation. Bold numbers indicate correlations with absolute value > 0.1

Table 3: Correlations between the top 29 predictors and the target, [a]: Point Biserial Correlation; [b]: Cramer's V correlation; [c]: Phi correlation.

Rank	Acronym	Description	Variable Type	Correlation
1	WB2	Age of woman	interval	0.16 [a]
2	wscore	Wealth index score	interval	-0.11 [a]
3	HC6	Type of fuel for cooking	ordinal	0.2 [b]
4	WS8	Type of toilet facility	nominal	0.2 [b]
5	CM6	Children not living with you	dichotomous	0.08 [c]
6	HC8E	Refrigerator	dichotomous	-0.09 [b]
7	HH11	Number of HH members	interval	0.07 [a]
8	hHighEL	Education of HH head	ordinal	0.16 [b]
9	WS1	Main source of drinking water	nominal	0.19 [b]
10	CM4	Children living with you	dichotomous	-0.04 [c]
11	WB3	Ever attended school	dichotomous	-0.08 [c]
12	HW1	Place for hand washing	nominal	0.16 [b]
13	HC8A	Electricity	dichotomous	-0.09 [c]
14	HH14	Number of children $<$ 5 years	interval	-0.01 [a]
15	HC8C	Television	dichotomous	-0.09 [c]
16	windex5	Wealth index quintiles	ordinal	0.1 [b]
17	HH12	Number of women 15 - 49 years	interval	0.03 [a]
18	DV1D	Beat Just: refuses sex	dichotomous	0.04 [c]
19	HC13	HH own any animals	dichotomous	0.06 [c]
20	HC10	HH owns the dwelling	dichotomous	0.03 [c]
21	HH6	Area	dichotomous	-0.06 [c]
22	DV1B	Beat Just: neglects children	dichotomous	0.04 [c]
23	DV1C	Beat Just: argues w/ husband	dichotomous	0.03 [c]
24	DV1A	Beat Just: goes out w/o tell	dichotomous	0.03 [c]
25	WS6	Treat water for drinking	dichotomous	0 [c]
26	HC8B	Radio	dichotomous	0 [c]
27	HHSEX	Sex of household head	dichotomous	0.01 [c]
28	HC9C	Bicycle	dichotomous	0.01 [c]
29	HC9A	Watch	dichotomous	-0.04 [c]

References

1. Zhang, Z. Multiple imputation with multivariate imputation by chained equation (MICE) package. *Annals of Translational Medicine* **2016**, *4*.
2. Buck, S.F. A method of estimation of missing values in multivariate data suitable for use with an electronic computer. *Journal of the Royal Statistical Society: Series B (Methodological)* **1960**, *22*, 302–306.
3. Pedregosa, F.; Varoquaux, G.; Gramfort, A.; Michel, V.; Thirion, B.; Grisel, O.; Blondel, M.; Prettenhofer, P.; Weiss, R.; Dubourg, V.; others. Scikit-learn: Machine learning in Python. *Journal of Machine Learning Research* **2011**, *12*, 2825–2830.
4. Breiman, L. Random forests. *Machine Learning* **2001**, *45*, 5–32.
5. Jurman, G.; Riccadonna, S.; Furlanello, C. A comparison of MCC and CEN error measures in multi-class prediction. *PloS one* **2012**, *7*.
6. Powers, D.M.W. Evaluation: From precision, recall and F-measure to ROC, informedness, markedness & correlation. *Journal of Machine Learning Technology* **2011**, *2*, 37–63.
7. Shi, L.; Campbell, G.; Jones, W.D.; Campagne, F.; Wen, Z.; Walker, S.J.; Su, Z.; Chu, T.M.; Goodsaid, F.M.; Pusztai, L.; others. The MicroArray Quality Control (MAQC)-II study of common practices for the development and validation of microarray-based predictive models. *Nature Biotechnology* **2010**, *28*, 827.
8. Jurman, G.; Riccadonna, S.; Visintainer, R.; Furlanello, C. Algebraic comparison of partial lists in bioinformatics. *PloS one* **2012**, *7*.
9. Huber, P.J. *Robust statistics*; Vol. 523, John Wiley & Sons, 2004.
